# The Late Dr. Cagney

**Published:** 1897-12-04

**Authors:** 


### THE LATE DR. CAGNEY.

It is with great regret that we record the death of Dr. James Cagney, who had made for himself a high and honourable position in the medical and social world in London. Dr. Cagney had made his mark more especially in nervous diseases, and no one who knew him could doubt that he had a bright future and a most useful career before him. At the time of his death he was attached to three hospitals, attendance at which entailed upon him a large amount of work. Outside his own immediate circle he was perhaps most widely known in connection with the Irish Medical Schools and Graduates' Association, and for his earnest efforts to remove what he always felt was a standing injustice to his fellow countrymen, viz., the ineligibility of Fellows of the Irish colleges for positions on the staffs of most English hospitals.

